# A case of podocytic infolding glomerulopathy with multiple myeloma

**DOI:** 10.1186/1471-2369-15-32

**Published:** 2014-02-13

**Authors:** Makoto Harada, Yuji Kamijo, Takashi Ehara, Hisashi Shimojo, Hidekazu Shigematsu, Makoto Higuchi

**Affiliations:** 1 Department of Nephrology, Shinshu University School of Medicine, 3-1-1, Asahi, Matsumoto, Nagano 390-8621, Japan; 2 Department of Pathology, Shinshu University School of Medicine, 3-1-1 Asahi, Matsumoto, Nagano 390-8621, Japan

**Keywords:** Podocytic infolding glomerulopathy, Multiple myeloma, Microspheres

## Abstract

**Background:**

Podocytic infolding glomerulopathy (PIG) is a recently described condition causing rare pathological changes to the glomeruli, and has attracted considerable attention. PIG is characterized by specific changes to the thickened glomerular basement membrane (GBM), including microspheres, microtubular structures, and podocytic infolding. Only a small number of cases of PIG have been reported. The clinical features and pathogenesis of this condition are still unclear. To elucidate the characteristics of this glomerulopathy, it is necessary to accumulate information from reported cases. We present here the first reported case of PIG with multiple myeloma.

**Case presentation:**

A 79-year-old Japanese man was admitted to his local hospital with proteinuria, hypergammaglobulinemia, hypoalbuminemia, and kidney dysfunction. Laboratory tests revealed monoclonal IgG(λ) M proteins in the serum and Bence-Jones proteins in the urine. Bone marrow aspiration showed monoclonal plasma cell proliferation, indicating a diagnosis of multiple myeloma. Renal biopsy was performed to determine the cause of the proteinuria and kidney dysfunction. Histological examination of the biopsy specimen showed glomeruli with an irregularly thickened GBM and bubble-like structures in the capillary walls. Immunofluorescence staining did not show glomerular deposition of immunoglobulins, light chains, or complement components. Congo red staining did not show amyloid deposition. Electron microscopy showed an irregularly thickened GBM with unusual structures in the glomerular capillary walls including podocytic infolding and microspheres, suggesting PIG. There were no electron-dense deposits in the GBM, while various findings indicating podocyte injury were detected.

**Conclusion:**

We present here the first reported case of PIG in a patient with multiple myeloma. The mechanisms underlying the development of PIG in multiple myeloma are unknown, but may be associated with podocyte injury.

## Background

Podocytic infolding glomerulopathy (PIG) was recently described by Joh et al. [1] and has attracted considerable attention because of the characteristic pathological changes to the glomeruli. The glomerular changes are characterized by specific lesions of the thickened glomerular basement membrane (GBM) including microspheres, microtubular structures, and podocytic infolding [1]. Patients with PIG always present with proteinuria, and often have kidney dysfunction [1].

PIG is not included in the current World Health Organization classification of glomerular diseases. Only a small number of cases of PIG have been reported to date, and these have all been in Japan. These reports show that PIG tends to be associated with autoimmune abnormalities, such as systemic lupus erythematosus (SLE). Although some specialists consider that PIG should be classified as a new disease entity, it is also possible that PIG reflects a transient morphological change in patients with conditions such as SLE and membranous nephropathy. In addition, the clinical features and pathogenesis of PIG are still unclear. To elucidate these issues, it is important to accumulate information from reported cases. We present here the first reported case of PIG in a patient with multiple myeloma.

## Case presentation

**Figure 1:**
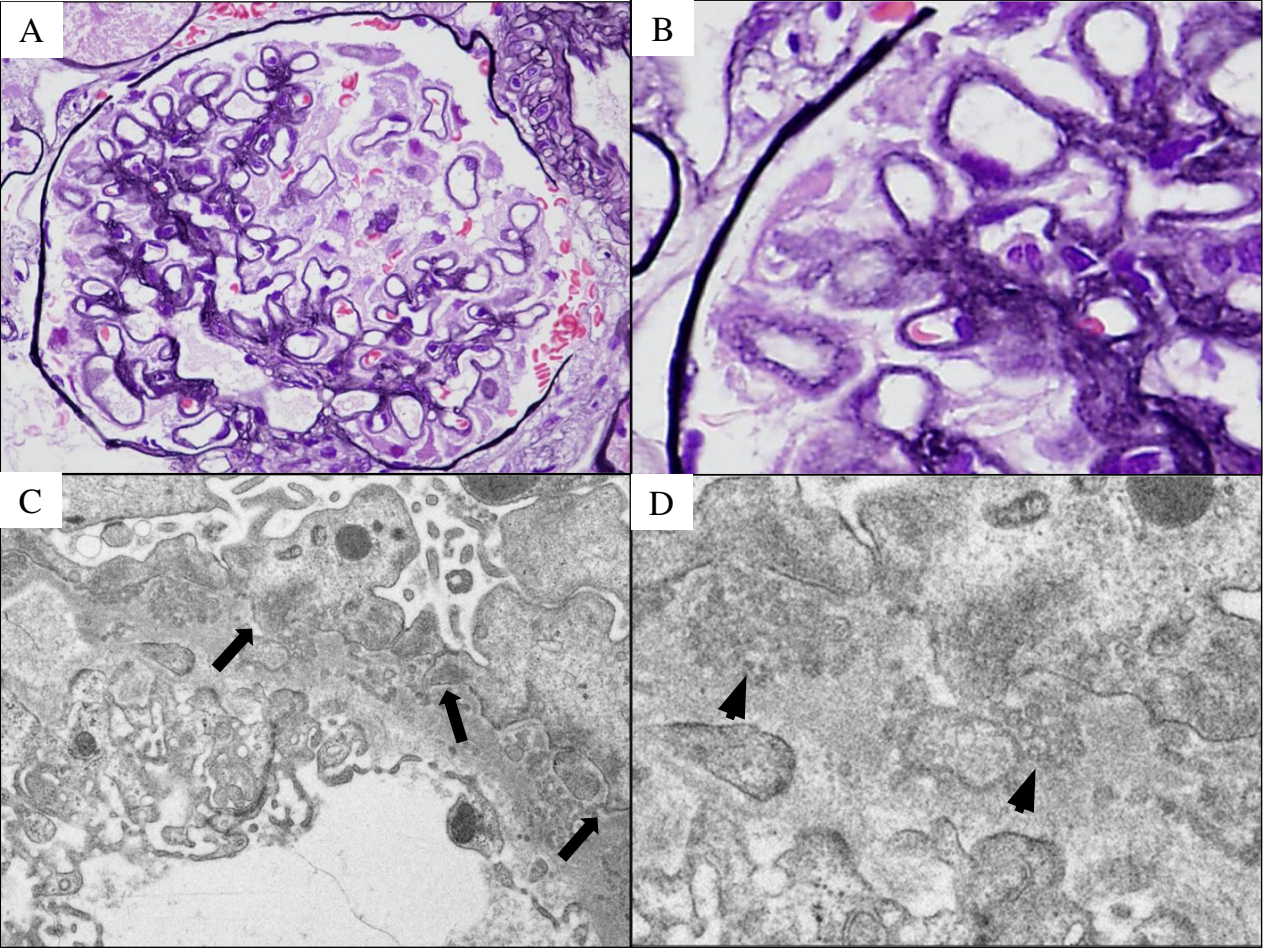
**Pathological examination findings of the renal biopsy sample. A**, **B**: Histological examination showed glomeruli with irregularly thickened glomerular basement membrane (GBM) with a bubble-like appearance of the capillary walls (periodic-acid methenamine silver staining). **C**, **D**: Electron microscopy showed unusual structures in the irregularly thickened GBM, including podocytic infolding (arrows) and microspheres (arrowheads), suggesting podocytic infolding glomerulopathy. Foot process flattening was observed with formation of microvilli and increased actin filaments in the foot processes. There were no dense deposits in the GBM.

A 79-year-old Japanese man presented with proteinuria, hypoalbuminemia, and increasing kidney dysfunction, and was admitted to his local hospital. He had a 3-year history of hypertension, hyperlipidemia, and hyperuricemia with mild kidney dysfunction (serum creatinine level 1.1 mg/dL at age 76 years). He had been treated with an angiotensin II receptor blocker, statin, and allopurinol for 3 years. His proteinuria and hypoalbuminemia had gradually worsened, with increasing serum creatinine levels. On admission, his blood pressure was 140/67 mmHg. Physical examination revealed no leg edema. Laboratory tests showed marked hypergammaglobulinemia with hypoalbuminemia (total protein 8.1 g/dL, albumin 3.3 g/dL), kidney dysfunction (blood urea nitrogen 28 mg/dL, serum creatinine 1.28 mg/dL), hyperuricemia (uric acid 9.8 mg/dL), high levels of beta-2 microglobulin (5.9 mg/L) and IgG (3076 mg/dL), and low levels of IgA (35 mg/dL) and IgM (24 mg/dL). Pancytopenia and autoimmune abnormalities, such as anti-nuclear antibody, rheumatoid factor, and hypocomplementemia, were not detected. Urinalysis showed proteinuria without hematuria (total urine protein and albumin excretion, 1423 and 949 mg/day, respectively), and a high concentration of a tubulointerstitial injury marker (N-acetyl-beta-D-glucosaminidase 35.9 U/L). Serum and urine immunofixation electrophoresis showed monoclonal IgG(λ) M proteins in the serum and Bence-Jones proteins in the urine. Bone marrow aspiration showed plasma cell proliferation (plasma cell count 22%). Considering these findings, the patient was diagnosed with multiple myeloma. A renal biopsy was performed to determine the cause of the proteinuria and kidney dysfunction. The biopsy specimen had 30 glomeruli, including one with global sclerosis and three with adhesive lesions. Histological examination showed glomeruli with an irregularly thickened GBM and a bubble-like appearance in the capillary walls (Figure 1A, B). Immunofluorescence and Congo red staining showed no glomerular deposition of immunoglobulins (IgG, IgA, IgM), light chains (κ, λ), complement components (C3, C4d, C1q), or amyloid protein. Surprisingly, electron microscopy showed podocytic infolding and microspheres in the irregularly thickened GBM (Figure 1C, D) as well as foot process flattening, with formation of microvilli and increased actin filaments in the foot processes (Figure 1C, D). There were no electron-dense deposits in the GBM. These pathological changes were similar to those of the glomerulopathy recently described by Joh et al. [1], suggesting a diagnosis of PIG. Although marked hyalinosis and severe intimal thickening of the renal interlobular arteries were not observed, there were focal tubulointerstitial lesions accompanied by tubular atrophy, as well as interstitial fibrosis and inflammatory cell infiltration, suggesting focal renal ischemic changes. However, there were no changes indicating cast nephropathy or amyloidosis, which are often associated with myeloma. The patient had many risk factors for atherosclerosis such as advanced age, hypertension, diabetes mellitus, hyperlipidemia, and hyperuricemia. Therefore, we considered that the increasing kidney dysfunction resulted from glomerular dysfunction due to PIG superimposed on underlying chronic renal ischemia. The patient was discharged and was treated with a moderate dose of corticosteroids (prednisolone 20 mg/day) after confirmation of the final pathological diagnosis. His proteinuria and his general condition slowly improved, but he died suddenly from a severe acute pulmonary infection 2 months after the introduction of steroid therapy.

## Discussion

PIG is a rare glomerulopathy characterized by unique pathological changes in the glomerular capillary wall, including podocytic infolding and microspheres in the GBM, as found in the current case. The mechanisms underlying the development of PIG are still unclear. Several previous studies using immune electron microscopy found deposition of complement components and/or complement C5b-C9 complexes in extracellular organized structures, which are morphologically similar to microspheres, in the GBM in various kinds of glomerular diseases [2,3]. Furthermore, Fujigaki et al. analyzed the glomerular lesions of PIG using immune electron microscopy, and detected complement C5b-C9 and vimentin (a component of podocytes) in extracellular organized structures in the GBM [4]. These pathological findings indicate an association between hyperactivation of the complement pathway and development of the characteristic microstructures of PIG. The largest clinical study of PIG, which included 25 patients, reported a high incidence of autoimmune disease (SLE in 14 cases, Sjögren’s syndrome in four cases, autoimmune thyroid disease in two cases, rheumatoid arthritis in one case, mixed connective tissue disease in one case, and primary biliary cirrhosis in one case), and reported that corticosteroid therapy reduced the proteinuria [1]. These findings suggest that immune abnormalities with hyperactivation of the complement pathway may play an important role in the pathogenesis of PIG. We present here the first reported case of PIG in a patient with multiple myeloma. Previous studies reported various functional abnormalities and hyperactivation of the complement pathway in patients with multiple myeloma [5-8]. Lugassy et al. reported that the terminal complement pathway activating C5b-C9, as well as the classical and alternative complement pathways, are activated in the early stage as well as the later stages of multiple myeloma [9]. These complement pathway abnormalities may induce PIG via a similar process to that causing PIG in autoimmune diseases. In the current case, however, we did not detect any autoimmune abnormalities or hypocomplementemia by routine laboratory examinations, and there was no glomerular deposition of complement components (C3, C4d, C1q). It is therefore possible that hyperactivation of the complement pathway did not play a significant role in the development of PIG in the current case, and that PIG may also result from other mechanisms.

Matsuo et al. reported a patient with podocytic infolding lesions associated with focal segmental glomerulosclerosis secondary to vesicoureteral reflux, suggesting that the podocytic infolding lesions were a reaction to podocyte injury [10]. They reported similar changes to those found in our patient, with various pathological changes in the foot process structures such as flattening, formation of microvilli, and increased actin filaments, suggesting podocyte injury. This suggests that podocyte injury may result in the development of PIG irrespective of complement activation. This new information may help to increase our understanding of the pathogenesis of PIG.

We considered the possibility that PIG is an unusual type of membranous nephropathy associated with microspherical particles that developed after resolution of immune complex deposition in the GBM. To evaluate this possibility, we compared the clinical characteristics of patients with PIG and idiopathic membranous nephropathy (IMN) in previously reported Japanese studies. We found that these two groups of patients had different background characteristics. The mean age at the time of diagnosis was 41.8 years in patients with PIG and 59 years in patients with IMN [1,11]. The male-to-female ratio was almost 1:3 in patients with PIG and almost 1:1 in patients with IMN [1,11]. The mean urinary protein excretion was 2.2 g/day in patients with PIG and 7.9 g/day in patients with IMN [1,11]. The therapeutic responses also appeared to vary between these two groups of patients. All reported cases of nephrotic PIG associated with autoimmune disease achieved complete remission with corticosteroid therapy, whereas only 66.7% of cases of nephrotic IMN achieved complete or incomplete remission with corticosteroid therapy [1,12]. Although spontaneous remission may occur in some cases of IMN, complete or incomplete remission did not occur in any of the reported cases of nephrotic PIG not associated with autoimmune disease and not treated with corticosteroids [1]. These findings suggest that PIG is strongly influenced by corticosteroid therapy and by associated autoimmune disease, and that the response to therapeutic intervention differs between patients with PIG and IMN. We consider these findings to indicate that PIG is a new disease entity that differs from IMN.

## Conclusion

We present here the first reported case of PIG in a patient with multiple myeloma. Although the pathogenesis of PIG is still unclear, our findings suggest that podocyte injury may result in PIG. Further case reports should be accumulated to increase our understanding of PIG.

## Consent

The present study complied with the guidelines of the 2008 revision of the Helsinki Declaration. The institutional review board of Shinshu University approved use of the patient’s medical records and images for this report (approval No. 884). Written informed consent was obtained from the patient for publication of this Case report and any accompanying images, prior to kidney biopsy. A copy of the written consent is available for review by the Editor of this journal.

## Abbreviations

GBM: Glomerular basement membrane; PIG: Podocytic infolding glomerulopathy; SLE: Systemic lupus erythematosus; C5b-9: Complement 5b-9; IMN: Idiopathic membranous nephropathy.

## Competing interests

The authors declare that they have no competing interests.

## Authors’ contributions

MH, YK, and MH treated the patient. MH drafted the manuscript. YK and MH helped to draft the manuscript. HS, TE, and HS performed the pathological analyses. All the authors contributed to preparation of the manuscript, and approved the final version.

## Pre-publication history

The pre-publication history for this paper can be accessed here:

http://www.biomedcentral.com/1471-2369/15/32/prepub
